# No association between HPV vaccine and reported post-vaccination symptoms in Japanese young women: Results of the Nagoya study

**DOI:** 10.1016/j.pvr.2018.02.002

**Published:** 2018-02-23

**Authors:** Sadao Suzuki, Akihiro Hosono

**Affiliations:** Department of Public Health, Graduate School of Medical Sciences, Nagoya City University, 1 Kawasumi, Mizuho-cho, Mizuho-ku, Nagoya 466–8601, Japan

**Keywords:** Human papilloma virus vaccine, Post-vaccination symptom, Population based epidemiological study, Questionnaire

## Abstract

Nagoya City introduced free HPV vaccination in 2010 and in April 2013 the Ministry of Health, Labour and Welfare included the HPV vaccine in the National Immunization Program. However, in June 2013, the Ministry suspended proactive recommendation of the vaccine after unconfirmed reports of adverse events. To investigate any potential association between the vaccine and reported symptoms, Nagoya City conducted a questionnaire-based survey.

Participants were 71,177 female residents of Nagoya City born between April 2, 1994 and April 1, 2001. The anonymous postal questionnaire investigated the onset of 24 symptoms (primary outcome), associated hospital visits, frequency, and influence on school attendance.

Totally, 29,846 residents responded. No significant increase in occurrence of any of the 24 reported post-HPV vaccination symptoms was found. The vaccine was associated with increased age-adjusted odds of hospital visits for “abnormal amount of menstrual bleeding” (OR: 1.43, 95% CI: 1.13–1.82), “irregular menstruation” (OR: 1.29, 95% CI: 1.12–1.49), “severe headaches” (OR: 1.19, 95% CI: 1.02–1.39), and chronic, persisting “abnormal amount of menstrual bleeding” (OR 1.41, 95% CI: 1.11–1.79). No symptoms significantly influenced school attendance and no accumulation of symptoms was observed.

The results suggest no causal association between the HPV vaccines and reported symptoms.

## Highlights

• A large-scale epidemiological study on HPV vaccination and reported post vaccination symptoms was performed.
• Age-adjusted analyses found no significant association between HPV vaccines and occurrence of the 24 reported symptoms.
• Increased odds of hospital visits for some symptoms is unlikely to be due to any biological association.

## 1 Introduction

Cervical cancer is the fourth most common cancer among women worldwide, with about 528,000 new cases and 266,000 deaths in 2012 [1]. In Japan, mortality from cervical cancer is increasing in women of reproductive age [2], [3]. Furthermore, between 1994 and 2011, there was a 4.4% annual percentage increase in incidence of cervical cancer in women aged 15–39 years [4]. Most women who develop cervical cancer are either under-screened or never-screened. The cervical cancer screening rate in Europe and Northern America, is roughly 80%; however, in Japan it was only 37.7% in 2011 [5].

The World Health Organization (WHO) recommends human papillomavirus (HPV) vaccination to prevent HPV infection and the development of cervical cancer and other HPV-related diseases [6]. The bivalent HPV vaccine was licensed in Japan in October 2009. Soon after this, Nagoya City, with a population of approximately 2,300,000 and located in the center of Japan, initiated a fully financed HPV vaccination program to encourage vaccination of girls born between April 2, 1996 and April 1, 1998 and in the first and second year of junior high school. In April 2013, HPV was added to the list of recommended routine vaccinations in Japan. However, following unconfirmed reports of unusual post-vaccination symptoms, the Japanese Ministry of Health, Labour and Welfare (MHLW) suspended its proactive recommendations for the vaccine in June 2013 [7] and instructed local health authorities to stop promoting the use of the vaccine until the suspected adverse events had been investigated. This led to a rapid and dramatic decrease in vaccination coverage, even though the HPV vaccines were freely available for the target age-group [8], [9].

Epidemiological studies conducted globally since the launch of the HPV vaccines have failed to find any increased risk of developing conditions claimed to be associated with the vaccine such as complex regional pain syndrome (CRPS) [10], [11], postural orthostatic tachycardia syndrome (POTS) [11], multiple sclerosis or other demyelinating diseases [12], venous thromboembolism [13], or any other adverse events following immunization [14]. A meta-analysis [15] and a recent review [16] found that both the bi- and quadrivalent HPV vaccines were safe. Descriptive studies such as case reports of CRPS, POTS [17] and a novel disease entity, HPV vaccination-associated neuro-immunopathetic syndrome (HANS) [18], have been published in Japan. However, no analytical epidemiological studies investigating the association between HPV vaccines and reported post-vaccination symptoms, have been published.

In January 2014, following clinical evaluation of individual cases, the MHLW concluded that the reported post-vaccination symptoms were not causally associated with HPV vaccination but were psychosomatic responses [19]. Despite this statement, the MHLW insisted that further studies were required before once again promoting the use of HPV vaccines [20]. It has been argued that this decision was not based on adequate scientific evidence because the Japanese vaccination system suffers from a failure of governance [21]. The decision to suspend proactive recommendations for the HPV vaccines traveled globally through online networks and social media and was applauded by antivaccination groups but not by the global scientific community [22]. In August 2015, the Japan Society of Obstetrics and Gynecology demanded the resumption of proactive recommendations for HPV vaccination because it is recommended for the prevention of cervical cancer [23]. Others also argued that a fair and transparent policy, including no-fault compensation, was required when implementing a new vaccination program, including the HPV vaccine, in Japan [24], [25]. Furthermore, in December 2015, the WHO Global Advisory Committee on Vaccine Safety (GACVS) noted in their Statement on the Safety of HPV Vaccines that in Japan “young women are being left vulnerable to HPV-related cancers that otherwise could be prevented. As GACVS has noted previously, policy decisions based on weak evidence, leading to lack of use of safe and effective vaccines, can result in real harm” [26]

In March 2014, Nagoya City council requested that the national government investigate any possible causal association between HPV vaccines and reported post-vaccination symptoms, and to recommend treatments for such symptoms. In January 2015, the Aichi Branch of All Japan Coordinating Association of HPV Vaccine Sufferers asked for and received approval from the Mayor of Nagoya City for an investigation into reported symptoms after HPV vaccination. Consequently, Nagoya City and the Nagoya City University agreed to conduct a large-scale questionnaire-based study in April 2015 to investigate any association between HPV vaccines and reported post-vaccination symptoms. The results of this study are presented in this paper

## 2 Methods

Participants eligible for the study were women born between April 2, 1994 and April 1, 2001 (9–15 years old on April 1, 2010), who were residents of Nagoya City as of August 12, 2015. The survey tool was an anonymous postal questionnaire. The study protocol was reviewed and approved by the Ethics Committee of the Nagoya City University, Graduate School of Medical Sciences (approval number 1206).

The anonymous questionnaire was mailed to each eligible resident in September 1, 2015; responses that were returned before November 2, 2015 were evaluated. The responses included information on the birth date of the person eligible for vaccination, which was stratified by year to one of seven 1-year age groups (categories) beginning on April 2, 1994 and ending on April 1, 1995 through 2001, and information on the responder (the person eligible for vaccination, the person eligible for vaccination with help from her parent or caregiver, or only the parent or caregiver). The onset of one or more of 24 symptoms between the sixth year of elementary school and the time of the survey were the primary outcomes. These included: irregular menstrual periods; abnormal amount of menstrual bleeding; pain in the joints or other parts of the body; severe headache; fatigue; poor endurance; difficulty concentrating; visual field abnormalities; abnormal sensitivity to light; sudden vision loss; dizziness; cold feet; difficulty falling asleep; abnormally long sleep duration; skin problems; hyperventilation; memory decline;, loss of ability to perform simple calculations; loss of ability to remember fundamental Kanji [Chinese characters indispensable for life in Japan]; involuntary uncontrollable body movements; loss of the ability to walk normally; becoming dependent on a walking stick or wheelchair; sudden loss of strength; and weakness in the hands and feet. The month and year of symptom onset, hospital visits because of symptoms, current frequency of the symptoms (i.e., always, sometimes, rarely, or never), influence of symptoms on school attendance or employment, HPV vaccination history (the number of injections and the injection schedule), type of HPV vaccine: the bivalent HPV vaccine (Cervarix, GlaxoSmithKline) or the quadrivalent HPV vaccine (Gardasil, Merck & Co.), and reasons for not completing the full immunization schedule, if applicable, were recorded. Items to be included in the questionnaire items were selected after consultations with both the study investigators and the Aichi Branch of the All Japan Coordinating Association of HPV Vaccine Sufferers. The original Japanese version of the questionnaire [27] and an English language translation [28] are available online.

### 2.1 Statistical analysis

Association of the HPV vaccination with one or more of the 24 reported symptoms was the primary outcome. This was evaluated in the entire study population. Other outcomes such as hospital visits for symptoms, persistent and constant symptoms (answered “always” for current frequency of the symptoms), influence on life activities, and occurrence of multiple symptoms, were also investigated for any potential association with HPV vaccination. Logistic regression analysis was used to calculate age-adjusted odds ratios (ORs) and 95% CIs. The analysis was repeated for each vaccine individually (Cervarix or Gardasil).

**Fig. 1:**
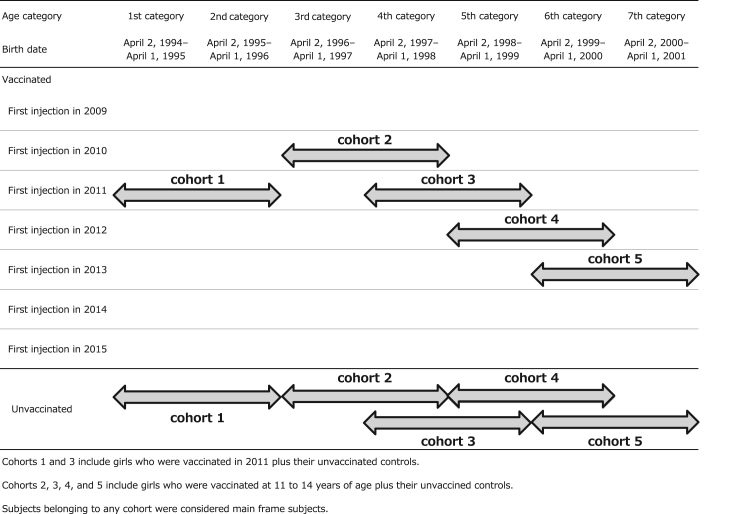
Subjects Included in the Five Main Frame Cohorts of the Subgroup Analyses. Cohorts 1 and 3 include girls who were vaccinated in 2011 plus their unvaccinated controls. Cohorts 2, 3, 4, and 5 include girls who were vaccinated at 11–14 years of age plus their unvaccined controls. Subjects belonging to any cohort were considered main frame subjects.

Subgroup analyses by age and year of first injection (subgroup analysis I), and after exclusion of subjects with early-onset symptoms (subgroup analysis II), were also conducted. In both subgroups, subjects in each age category were selected by year of first vaccine injection and grouped to form 2-year age distribution as follows: first and second category in 2011; third category in 2010, fourth category in 2010 and 2011, fifth category in 2011 and 2012, sixth category in 2012 and 2013, and seventh category in 2013, as shown in Fig. 1 (also numbered in **bold** in Table 1). The entire population of vaccinated and unvaccinated subjects, grouped as described above, comprised the “main frame” of the study. For subgroup analysis I, five cohorts based on the year of the first injection were created. Each cohort included individuals born within two sequential years and comprising most vaccine recipients in the corresponding years. Girls of the same age who had not been vaccinated were also included in each cohort as controls. The structure of the cohorts is shown in Fig. 1. For example, cohort 3 consists of 1500 + 1300 vaccinated girls and 663 + 1260 unvaccinated ones (see Table 1). In the subgroup analysis I, two comparisons were performed; in terms of age difference between cohort 1 and 3, (fixed injection year of 2010) and time trend among cohort 2, 3, 4, and 5 (fixed age in 11–14 years old).

**Table 1:** Birth date, age category, and vaccination status of the study participants.

Vaccine status	Age category and birth date							Total
	1st category, April 2, 1994-April 1, 1995	2nd category, April 2, 1995-April 1, 1996	3rd category, April 2, 1996-April 1, 1997	4th category, April 2, 1997-April 1, 1998	5th category, April 2, 1998-April 1, 1999	6th category, April 2, 1999-April 1, 2000	7th category, April 2, 2000-April 1, 2001	
First injection (year)								
2009	98	48	80	27	9	2	0	264
2010	265	199	**2057**	**1239**	40	4	1	3805
2011	**2344**	**2629**	835	**1500**	**1300**	39	5	8652
2012	93	180	173	400	**1251**	**1354**	40	3491
2013	20	29	23	86	216	**495**	**507**	1376
2014	5	4	3	4	15	24	29	84
2015	5	1	1	0	5	10	7	29
Year unknown	735	659	553	510	322	195	73	3047
Number of injections								
1	184	133	117	106	127	196	255	1118
2	192	207	185	207	278	380	85	1534
3	3014	3256	3280	3296	2650	1476	296	17,268
Number unknown	175	153	143	157	103	71	26	828
Total vaccinated	3565	3749	3725	3766	3158	2123	662	20,748
Total unvaccinated	**496**	**428**	**452**	**663**	**1260**	**2038**	**3761**	9098
Vaccination coverage	87.8%	89.8%	89.2%	85.0%	71.5%	51.0%	15.0%	69.5%
Total participants	4061	4177	4177	4429	4418	4161	4423	29,846

Participants shown in **bold** were selected for the main-frame sub-group analyses.

**Fig. 2:**
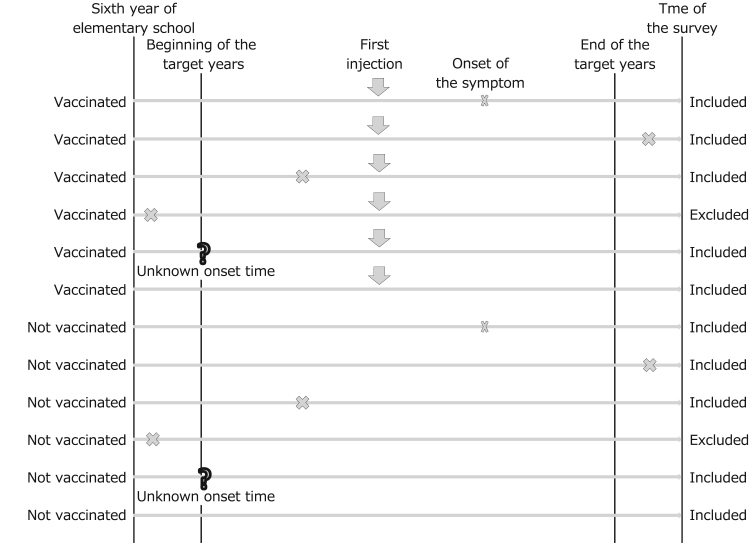
Subjects for subgroup analysis excluding symptoms that had occurred before the earliest target year of HPV vaccination.

Subgroup analysis II was also performed using data obtained from the main frame participants. Those with symptoms that had occurred before the year of the first HPV vaccination (i.e., not caused by the vaccine) were excluded (Fig. 2). The purpose of subgroup analysis II was to allow for analyses after excluding “noise” attributable to the grand analysis. This exclusion, performed regardless of vaccination status, prevented biased results from consideration of symptoms in vaccinated girls before they received the first injection. The statistical analysis was performed using SAS version 9.4 (SAS Institute).

## 3 Results

A total of 71,177 questionnaires were sent, 217 were returned as not deliverable, and 30,793 (43.4%) of the 70,960 distributed copies were returned by the recipients. Of those, 947 were excluded because of missing data on HPV vaccination status and/or age. The responses in the remaining 29,846 questionnaires were included in the analysis. Table 1 shows the birth-year distribution of participants, vaccination status including year of dose one, and the overall number of doses administered. Vaccination coverage in the first to fourth category was >85%. It began to decline in the fifth age category and dropped to 15% in the seventh category.

Table 2 shows the distribution of HPV vaccination status and occurrence of the 24 reported symptoms. The most common symptom was menstrual irregularity (26.3%) followed by cold feet (12.3%). Another six symptoms: severe headache, fatigue, poor endurance, dizziness, abnormally long duration of sleep, and skin problems were reported by > 10% of participants. In contrast, four symptoms: unable to perform simple calculations; involuntary uncontrollable body movements; loss of ability to walk in a normal way; and becoming dependent on a walking stick or wheelchair was reported by < 1.0% of participants.

**Table 2:** Distribution of vaccination status and occurrence of 24 symptoms.

Symptom		Vaccine (+)			Vaccine (−)			Total	
		Symptom (+)	Symptom (−)	Probability (％)	Symptom (+)	Symptom (−)	Probability (％)	Symptom unknown	Probability (％)
1	Menstrual irregularity	5468	15,138	26.5%	2310	6696	25.6%	234	26.3%
2	Abnormal amounts of menstrual bleeding	1625	18,946	7.9%	561	8434	6.2%	280	7.4%
3	Pain in the joints or other parts of the body	1507	19,071	7.3%	720	8276	8.0%	272	7.5%
4	Severe headache	2150	18,464	10.4%	925	8097	10.3%	210	10.4%
5	Fatigue	2268	18,342	11.0%	1037	7984	11.5%	215	11.2%
6	Poor endurance	2261	18,344	11.0%	991	8028	11.0%	222	11.0%
7	Difficulty concentrating	1430	19,159	6.9%	723	8294	8.0%	240	7.3%
8	Abnormal field of vision	389	20,193	1.9%	172	8845	1.9%	247	1.9%
9	Abnormal sensitivity to light	907	19,696	4.4%	356	8662	3.9%	225	4.3%
10	Sudden vision loss	1381	19,210	6.7%	795	8221	8.8%	239	7.3%
11	Dizziness	2282	18,315	11.1%	1089	7927	12.1%	233	11.4%
12	Cold feet	2508	18,077	12.2%	1144	7873	12.7%	244	12.3%
13	Difficulty falling asleep	1483	19,118	7.2%	692	8320	7.7%	233	7.3%
14	Abnormally long duration of sleep	2454	18,119	11.9%	1058	7955	11.7%	260	11.9%
15	Skin problems	2062	18,538	10.0%	1062	7950	11.8%	234	10.5%
16	Hyperventilation	700	19,913	3.4%	336	8694	3.7%	203	3.5%
17	Memory decline	623	19,992	3.0%	217	8805	2.4%	209	2.8%
18	Loss of ability to perform simple calculations	189	20,422	0.9%	79	8940	0.9%	216	0.9%
19	Loss of ability to remember fundamental Kanji	416	20,196	2.0%	181	8846	2.0%	207	2.0%
20	Involuntary uncontrollable body movements	201	20,413	1.0%	58	8964	0.6%	210	0.9%
21	Loss of ability to walk in a normal way	72	20,534	0.3%	22	8990	0.2%	228	0.3%
22	Becoming dependent on a walking stick or wheelchair	31	20,577	0.2%	16	8994	0.2%	228	0.2%
23	Sudden loss of strength	283	20,311	1.4%	100	8909	1.1%	243	1.3%
24	Weakness in the hands and feet	354	20,189	1.7%	124	8862	1.4%	317	1.6%

In our analyses, we hypothesized age and the person responding to the questionnaire (child versus parent) might be potential confounders. Age, but not responder, confounded the association between HPV vaccination and reported symptoms, therefore we decided all analyses should be age-adjusted. Table 3 shows the age-adjusted ORs for the association between HPV vaccination and occurrence of the 24 reported symptoms (primary outcomes), hospital visits for the symptoms, and persistent or continuing symptoms. None of the 24 reported symptoms were significantly associated with increased odds of occurring after administration of the HPV vaccine. Three symptoms had significantly increased odds of attending hospital: 1.43 (95% CI: 1.13–1.82) for “abnormal amount of menstrual bleeding”; 1.29 (95% CI: 1.12–1.49) for “irregular menstruation”, and 1.19 (95% CI: 1.02–1.39) for “severe headache”. Persistent occurrence of “abnormal amounts of menstrual bleeding” was also statistically significant, OR of 1.41 (95% CI: 1.11–1.79).

**Table 3:** Age-adjusted odds ratios of the association of vaccination and the occurrence of symptoms, hospital visits, and current symptoms.

Symptom		Occurrence of the symptom (main outcome)		Hospital visits		Persistent and constant symptom	
		OR	95％ CI	OR	95％ CI	OR	95％ CI
1	Menstrual irregularity	0.92	(0.86–0.98)	**1.29**	**(1.12–1.49)**	1.10	(0.97–1.24)
2	Abnormal amounts of menstrual bleeding	1.10	(0.98–1.23)	**1.43**	**(1.13–1.82)**	**1.41**	**(1.11–1.79)**
3	Pain in the joints or other parts of the body	0.87	(0.78–0.97)	1.25	(1.00–1.56)	0.71	(0.55–0.91)
4	Severe headache	0.95	(0.87–1.05)	**1.19**	**(1.02–1.39)**	1.08	(0.81–1.43)
5	Fatigue	0.81	(0.74–0.89)	1.28	(1.00–1.64)	0.83	(0.68–1.00)
6	Poor endurance	0.88	(0.81–0.97)	1.20	(0.91–1.58)	0.97	(0.81–1.15)
7	Difficulty concentrating	0.84	(0.76–0.94)	1.29	(0.89–1.88)	0.96	(0.77–1.20)
8	Abnormal field of vision	0.82	(0.67–1.01)	0.97	(0.64–1.47)	0.80	(0.45–1.44)
9	Abnormal sensitivity to light	0.98	(0.85–1.13)	1.03	(0.73–1.44)	0.98	(0.72–1.34)
10	Sudden vision loss	0.78	(0.70–0.87)	0.90	(0.79–1.03)	1.03	(0.83–1.29)
11	Dizziness	0.84	(0.77–0.92)	1.12	(0.92–1.37)	0.96	(0.74–1.25)
12	Cold feet	0.79	(0.73–0.87)	1.02	(0.66–1.57)	0.91	(0.79–1.05)
13	Difficulty falling asleep	.71	(0.64–0.79)	0.87	(0.65–1.19)	0.75	(0.60–0.93)
14	Abnormally long duration of sleep	.91	(0.83–0.99)	1.12	(0.78–1.60)	1.12	(0.95–1.33)
15	Skin problems	0.78	(0.71–0.85)	0.88	(0.79–0.99)	0.87	(0.75–1.00)
16	Hyperventilation	0.77	(0.66–0.90)	0.82	(0.63–1.07)	0.31	(0.10–0.91)
17	Memory decline	1.00	(0.84–1.19)	1.06	(0.55–2.06)	0.74	(0.53–1.02)
18	Loss of ability to perform simple calculations	0.70	(0.52–0.94)	1.83	(0.57–5.96)	0.35	(0.21–0.58)
19	Loss of ability to remember fundamental Kanji	0.73	(0.60–0.89)	2.09	(0.66–6.63)	0.44	(0.27–0.72)
20	Involuntary uncontrollable body movements	1.20	(0.87–1.66)	1.08	(0.56–2.07)	0.81	(0.32–2.07)
21	Loss of ability to walk in a normal way	0.94	(0.56–1.60)	1.21	(0.61–2.39)	0.42	(0.15–1.21)
22	Becoming dependent on a walking stick or wheelchair	0.55	(0.28–1.09)	0.57	(0.24–1.34)	0.36	(0.11–1.25)
23	Sudden loss of strength	1.05	(0.81–1.36)	1.41	(0.73–2.73)	0.59	(0.15–2.26)
24	Weakness in the hands and feet	1.19	(0.94–1.50)	1.42	(0.86–2.35)	1.02	(0.37–2.79)

Significant odds ratios are shown in **bold**.

Abbreviation: OR, odds ratio; CI, confidence interval.

There was no statistically significant association between any of the 24 reported symptoms and school performance, school activities other than studying, and job hunting and employment at 0.82 (95% CI: 0.74–0.91), 1.02 (95% CI: 0.89–1.15), and 0.71 (95% CI: 0.53–0.97), respectively. The ORs for development of one or more symptoms (0.83, 95% CI: 0.78–0.88), and >1 (0.81, 95% CI: 0.76–0.87), >2 (0.80, 95% CI: 0.75–0.86), >3 (0.79, 95% CI: 0.73–0.86), >4, (0.77, 95% CI: 0.70–0.84), and >9 (or '10 or more'). symptoms (0.76, 95% CI: 0.63–0.93), decreased, indicating no associated with HPV vaccination and multiple symptoms.

Because the bivalent vaccines was licensed two years before quadrivalent vaccine, 87.8% of recipients were vaccinated with the bivalent vaccine in 2010 and 2011 and 80.0% of recipients with the quadrivalent vaccine in 2012 and 2013. None of the ORs for association of symptoms after vaccination with the bivalent vaccine were significant, whereas the OR for experiencing “involuntary uncontrollable body movements” was significantly increased after vaccination with the quadrivalent vaccine (1.54, 95% CI: 1.04–2.29). However, the ORs for a hospital visit (1.19, 95% CI: 0.50–2.84), and persistent and constant symptom (0.52, 95% CI: 0.11–2.44), were not significant. No other symptoms were significantly associated with the quadrivalent vaccine.

The analyses were repeatedly performed to evaluate main frame data for each year of being administered the first dose and in the unvaccinated controls. The 23,774 participants included 14,676 vaccinated and 9098 unvaccinated girls shown in bold numbers in Table 1, and comprised 79.7% of the entire sample of 29,846 participants. We obtained almost identical results (data not shown) with those of the grand analysis shown in Table 3.

As shown in Table 4, the five cohorts differed in age and year of receiving the first dose of the HPV vaccine. Table 5 shows the age-adjusted ORs of the association between individual reported symptoms and the year of first vaccination. The ORs for occurrence of each of the 24 reported symptoms in cohorts 1 and 3 were all higher in cohort 3 participants compared to those in the older cohort 1 participants. No significant ORs were observed. Comparisons of cohorts 2, 3, 4, and 5 revealed increases in ORs in more recent years of vaccination. Although significant, high ORs were observed in cohorts 4 and 5, the involved symptoms differed in the two groups.

**Table 4:** Characteristics of the five age and immunization year cohorts for subgroup analysis.

Birth period	Cohort 1	Cohort 2	Cohort 3	Cohort 4	Cohort 5
	April 2, 1994-April 1, 1996	April 2, 1996-April 1, 1998	April 2, 1997-April 1, 1999	April 2, 1998-April 1, 2000	April 2, 1999-April 1, 2001
Maximum difference of birth date within the cohort	2 years	2 years	2 years	2 years	2 years
First injection (for vaccinated girls only)	2011	2010	2011	2012	2013
Age at the year of first injection	14–17 years old	11–14 years old	11–14 years old	11–14 years old	11–14 years old
Number of vaccinated girls	4973	3296	2800	2605	1002
Number of unvaccinated girls	0924	1115	1923	3298	5799
Total number	5897	4411	4723	5903	6801
Vaccination coverage (%)	84.3%	74.7%	59.3%	44.1%	14.7%
Comparison of age at first injection	√		√		
Comparison of year of first injection		√	√	√	√

**Table 5:** Age-adjusted odds ratio of association of individual symptoms and year of first vaccination.

Symptom		Cohort 1 first vaccination 2011		Cohort 2 first vaccination 2010		Cohort 3 first vaccination 2011		Cohort 4 first vaccination 2012		Cohort 5 first vaccination 2013	
		OR	95％ CI	OR	95％ CI	OR	95％ CI	OR	95％ CI	OR	95％ CI
1	Menstrual irregularity	0.74	(0.64–0.86)	1.01	(0.86–1.18)	0.90	(0.79–1.03)	0.95	(0.84–1.07)	1.09	(0.93–1.27)
2	Abnormal amounts of menstrual bleeding	0.90	(0.70–1.15)	1.30	(0.99–1.71)	1.01	(0.80–1.28)	1.03	(0.83–1.27)	1.09	(0.82–1.45)
3	Pain in the joints or other parts of the body	0.56	(0.45–0.70)	0.77	(0.60–0.99)	0.80	(0.63–1.00)	0.94	(0.77–1.16)	1.00	(0.77–1.30)
4	Severe headache	0.65	(0.53–0.80)	0.94	(0.76–1.17)	0.94	(0.78–1.14)	0.87	(0.73–1.04)	0.97	(0.77–1.23)
5	Fatigue	0.55	(0.45–0.66)	0.77	(0.62–0.95)	0.63	(0.52–0.76)	0.88	(0.75–1.04)	0.97	(0.77–1.22)
6	Poor endurance	0.60	(0.49–0.73)	0.89	(0.71–1.10)	0.78	(0.64–0.94)	0.91	(0.77–1.08)	0.96	(0.76–1.21)
7	Difficulty concentrating	0.59	(0.47–0.75)	0.77	(0.59–0.99)	0.68	(0.55–0.85)	0.91	(0.75–1.10)	0.87	(0.66–1.15)
8	Abnormal field of vision	0.49	(0.33–0.73)	0.71	(0.44–1.13)	1.02	(0.66–1.58)	0.91	(0.58–1.42)	1.35	(0.83–2.22)
9	Abnormal sensitivity to light	0.51	(0.38–0.68)	0.99	(0.71–1.39)	1.09	(0.82–1.47)	1.00	(0.76–1.33)	1.05	(0.72–1.53)
10	Sudden vision loss	0.51	(0.40–0.65)	0.91	(0.70–1.19)	0.91	(0.72–1.14)	0.94	(0.78–1.14)	0.61	(0.46–0.81)
11	Dizziness	0.64	(0.52–0.78)	0.73	(0.59–0.91)	0.79	(0.66–0.95)	0.84	(0.71–0.99)	1.00	(0.81–1.25)
12	Cold feet	0.68	(0.56–0.82)	0.76	(0.62–0.92)	0.73	(0.61–0.87)	0.90	(0.76–1.05)	0.74	(0.58–0.94)
13	Difficulty falling asleep	.43	(0.35–0.53)	0.66	(0.51–0.86)	0.85	(0.67–1.08)	0.81	(0.64–1.01)	0.96	(0.72–1.28)
14	Abnormally long duration of sleep	.66	(0.55–0.80)	0.94	(0.76–1.16)	0.89	(0.74–1.08)	0.94	(0.80–1.11)	0.87	(0.70–1.10)
15	Skin problems	0.72	(0.59–0.89)	0.68	(0.55–0.84)	0.75	(0.62–0.91)	0.86	(0.72–1.02)	0.90	(0.72–1.13)
16	Hyperventilation	0.39	(0.29–0.53)	0.93	(0.66–1.31)	0.74	(0.54–1.02)	0.77	(0.57–1.02)	0.93	(0.62–1.40)
17	Memory decline	0.49	(0.36–0.68)	0.78	(0.52–1.18)	0.81	(0.57–1.15)	1.19	(0.84–1.68)	**2.17**	**(1.43–3.30)**
18	Loss of ability to perform simple calculations	0.38	(0.23–0.62)	0.46	(0.24–0.87)	0.56	(0.30–1.02)	1.16	(0.58–2.31)	1.97	(0.89–4.39)
19	Loss of ability to remember fundamental Kanji	0.32	(0.23–0.46)	0.58	(0.37–0.90)	0.69	(0.43–1.09)	1.25	(0.80–1.95)	1.61	(0.99–2.62)
20	Involuntary uncontrollable body movement	0.56	(0.32–0.99)	1.78	(0.79–4.05)	1.27	(0.65–2.46)	1.64	(0.87–3.10)	1.51	(0.65–3.49)
21	Loss of ability to walk in a normal way	0.49	(0.19–1.26)	0.51	(0.19–1.39)	2.80	(0.61–13.01)	1.64	(0.44–6.16)	2.00	(0.54–7.48)
22	Becoming dependent on a walking stick or wheelchair	0.31	(0.09–1.07)	0.56	(0.14–2.21)	0.92	(0.15–5.68)	0.61	(0.11–3.35)	0.82	(0.10–6.77)
23	Sudden loss of strength	0.69	(0.42–1.12)	0.73	(0.40–1.30)	1.48	(0.81–2.71)	**1.67**	**(1.03–2.73)**	1.13	(0.57–2.24)
24	Weakness in the hands and feet	0.82	(0.49–1.35)	0.89	(0.51–1.55)	1.48	(0.92–2.37)	1.40	(0.93–2.12)	1.13	(0.63–2.02)

Significant odds ratios are shown in **bold**.

Abbreviation: OR, odds ratio; CI, confidence interval.

Table 6 shows the results of subgroup analysis II, wherein the subjects with symptoms that occurred before the first year of HPV vaccination were excluded. “Weakness of the hands and feet” was the only symptom with a significantly higher OR (1.44, 95% CI: 1.09–1.82). The ORs of the 24 reported symptoms that led to a hospital visit were higher than those in the grand analysis shown in Table 3. Five of the 13 symptoms that led to a hospital visit had significantly increased ORs of > 2.0. The highest OR was 6.15 (95% CI: 1.03–23.78) for “inability to remember fundamental Kanji”, followed by 4.59 (95% CI: 1.32–28.75) for “unable to do simple calculations”. There were only two hospital visits among unvaccinated subjects for these two symptoms.

**Table 6:** Age-adjusted odds ratios of association of vaccination with symptom, hospital visit, and current symptom after excluding subjects with early-onset symptoms.

Symptom		Occurrence of the symptom		Hospital visits		Persistent and constant symptom	
		OR	95％ CI	OR	95％ CI	OR	95％ CI
1	Menstrual irregularity	0.98	(0.91–1.06)	**1.34**	**(1.14–1.59)**	**1.18**	**(1.01–1.39)**
2	Abnormal amounts of menstrual bleeding	1.11	(0.97–1.27)	**1.54**	**(1.15–2.06)**	**1.54**	**(1.15–2.06)**
3	Pain in the joints or other parts of the body	0.84	(0.74–0.95)	**1.44**	**(1.09–1.90)**	0.68	(0.49–0.94)
4	Severe headache	0.98	(0.88–1.10)	**1.41**	**(1.16–1.72)**	1.22	(0.84–1.78)
5	Fatigue	0.82	(0.74–0.91)	**1.60**	**(1.19–2.15)**	0.92	(0.73–1.15)
6	Poor endurance	0.91	(0.82–1.00)	**1.64**	**(1.17–2.29)**	1.08	(0.88–1.32)
7	Difficulty concentrating	0.85	(0.75–0.96)	**1.71**	**(1.09–2.68)**	0.84	(0.64–1.09)
8	Abnormal field of vision	0.81	(0.64–1.03)	1.25	(0.77–2.02)	0.90	(0.43–1.89)
9	Abnormal sensitivity to light	0.95	(0.81–1.13)	1.19	(0.80–1.79)	0.95	(0.65–1.38)
10	Sudden vision loss	0.85	(0.75–0.97)	1.07	(0.90–1.27)	1.13	(0.88–1.47)
11	Dizziness	0.86	(0.78–0.96)	**1.33**	**(1.06–1.67)**	1.03	(0.76–1.41)
12	Cold feet	0.80	(0.72–0.88)	1.34	(0.78–2.29)	0.96	(0.80–1.16)
13	Difficulty falling asleep	.68	(0.60–0.76)	1.09	(0.75–1.58)	0.75	(0.58–0.98)
14	Abnormally long duration of sleep	.90	(0.81–0.99)	1.30	(0.85–1.99)	1.12	(0.92–1.37)
15	Skin problems	0.83	(0.75–0.92)	1.01	(0.88–1.16)	0.97	(0.81–1.15)
16	Hyperventilation	0.83	(0.69–0.99)	1.06	(0.76–1.47)	0.20	(0.04–0.87)
17	Memory decline	0.94	(0.77–1.14)	1.69	(0.75–3.77)	0.71	(0.49–1.01)
18	Loss of ability to do simple calculations	0.68	(0.49–0.95)	**4.95**	**(1.03–23.78)**	0.32	(0.18–0.56)
19	Loss of ability to remember fundamental Kanji	0.66	(0.53–0.82)	**6.15**	**(1.32–28.75)**	0.39	(0.22–0.67)
20	Involuntary uncontrollable body movement	1.40	(0.97–2.01)	1.99	(0.89–4.47)	1.12	(0.36–3.49)
21	Loss of ability to walk in a normal way	1.45	(0.75–2.82)	**2.65**	**(1.02–6.91)**	1.44	(0.23–8.99)
22	Becoming dependent on a walking stick or wheelchair	0.90	(0.37–2.22)	1.02	(0.30–3.52)	0.70	(0.14–3.41)
23	Sudden loss of strength	1.13	(0.85–1.50)	**2.61**	**(1.16–5.87)**	0.79	(0.17–3.63)
24	Weakness in the hands and feet	**1.41**	**(1.09–1.82)**	**2.00**	**(1.11–3.61)**	1.24	(0.42–3.68)

Significant odds ratios are shown in **bold**.

Abbreviation: OR, odds ratio; CI, confidence interval.

## 4 Discussion

To the best of our knowledge, this is the first large-scale epidemiological study to investigate any association between HPV vaccines and reported symptoms in Japanese young women by direct comparison within the population.

None of the age-adjusted ORs of the primary study outcomes in the main analysis, namely the 24 symptoms that have been reported in the media, by the victims’ supports group or proposed by some physicians as being associated with HPV vaccination were significant. The ORs of three symptoms that led to a hospital visit were significant. Of those, “abnormal amounts of menstrual bleeding” (OR = 1.43) was the only one that persisted at the time of the study and had a significantly high OR. No other symptoms that persisted at the time of the study had significantly higher ORs in the vaccinated group. Although the subgroup analyses revealed significantly increased ORs for several symptoms, none remained consistently high in all three analyses. There was also no evidence of the occurrence of multiple concurrent symptoms as reported in the Japanese media after HPV vaccination or by a few physicians. Significant findings were also absent for each vaccine when analyzed separately, although significantly higher ORs were occasionally observed in the secondary analyses. Additionally, the planned alpha error of 5%, should be considered when interpreting these results. Our findings support the reports of WHO and the Japanese Government regarding the safety of these HPV vaccines.

The influence of age at vaccination is apparent from the difference in ORs between cohorts 1 and 3. However, the lower ORs seen in cohort 1 could be attributed to the higher vaccination coverage in that group causing the relatively poor health conditions of the unvaccinated girls (frailty selection bias) in cohort 1 [29], [30]. However, the problem of the frailty bias might be less serious in the relatively homogeneous school population than in an aged population with various health conditions. Furthermore, HPV vaccination is not contraindicated for the reported post-vaccination symptoms. Therefore the presence of these symptoms would not be a reason not to vaccinate against HPV. As for the yearly trend in ORs, the number of symptoms with point estimates of ORs > 1 increased over time, i.e., 3, 7, 9, and 13 symptoms in cohorts 2, 3, 4, and 5, respectively. These increases may have occurred within a short 4-year period because of the relatively poor health status of girls in the unvaccinated groups in the early study period when the vaccination coverage was higher [30]. Other reasons include enhanced negative psychological effects in response to negative opinions about HPV vaccination and because memory of vaccine-associated symptoms faded as time passed. Marked changes in ORs over a short interval are unlikely to have a biological cause and suggest the involvement of socio-psychological factors and/or changes in the characteristics of subjects with vaccination coverage.

Exclusion of subjects with symptoms that occurred before the first vaccination year did not alter the ORs for the primary outcome of symptom occurrence, but it did increase the ORs of symptoms that led to a hospital visit compared with the grand (Table 3) and main frame analyses (not shown). After exclusion, the ORs of 13 of 24 symptoms that led to a hospital visit were significantly increased. This could have resulted from relatively more severe symptoms among HPV vaccine recipients which needed a physician's diagnosis/treatment (reason A), more frequent doctor visits by vaccine recipients because they were anxious about causal relationship between HPV vaccine and symptoms (reason B), or a strong impression of HPV vaccination which had unconsciously moved the memory of the symptom onset to after the vaccination (reason C). The “loss of ability to remember fundamental Kanji” and “loss of ability to perform simple calculations” high ORs (6.15 and 4.95, respectively), but exclusion of participants with symptoms that occurred early in the study period, did not affect the occurrence or persistence of these symptoms. As shown in Table 6, the ORs of symptoms that led to a hospital visit differed significantly from those of the other types of outcomes. This suggests that hospital visits were not driven by the severity of symptoms but rather by changes in the attitude toward seeking medical advice and/or an effect of vaccination on the questionnaire responses. It is noteworthy that biological causal association (reason A), influence of vaccination on hospital-visiting behavior (reason B), and influence of vaccination on the questionnaire answers (reason C) became indistinguishable when the relationship of HPV vaccination and hospital visits was analyzed, resulting in a high OR regardless of a causal association. In addition, the highest ORs of 6.15 and 4.95 were calculated on only two subjects among the unvaccinated subjects, therefore they are not robust.

The most important study strength of this study was the inclusion of both recipients and non-recipients of HPV vaccine within the same population. The comparison was performed internally and did not rely on external data. Another study strength was very little missing data of HPV vaccine status and occurrence of symptoms, which would have been frequent if the study had depended on data retrieved from medical records only. Additionally, as we surveyed participant experiences of vaccine injection and occurrence of symptoms over time, the data reflected incidence cases rather than prevalence cases, which allowed a clear description of causal inferences. Thus, we believe that the findings of this study significantly advance our understanding on the lack of causal associations between HPV vaccines and post-vaccination symptoms. We also believe that the response rate of 43.4% is a strength of this study, since it was set in an urban area. We believe it was due to great efforts by Nagoya City, which included sending reminder letters to all of subjects, public broadcast via TV/subway news, and notification by flyers and posters, and so on. Finally, this survey allowed the parent or caregiver to respond and not just the child, which was an effective strategy to obtain a higher response rate. If we did not allow parents or caregivers to respond, the response rate might have been 23.0% (16,347/70,960) lower.

However, the first major limitation of this study was also an imperfect response rate resulting in possible selection bias that may have affected the results [31]. The questionnaire was sent to all female residents of Nagoya City born in specific years, so the estimated association would be unbiased if the response rate was sufficiently high. However, restricting the evaluation only to responders with an insufficient response rate would lead to selection bias of HPV vaccine and occurrence of symptoms [32]. While we cannot assess the magnitude of the bias theoretically, we have a base population and were could calculate the response rate, which provides stronger validation of the results than a smaller and/or unavailable response rate. Furthermore, the results of the subgroup analysis without these answers from parents only was quite close to the main analysis, i.e., no significantly high OR (data not shown). The results might be robust if the response rate was higher.

A second limitation was that the study design did not solicit very rare symptoms. Different study designs (e.g., case-control or monitoring system [33]) are necessary to investigate extremely rare or severe symptoms, including serious adverse events [34]. Insofar as mild or moderate symptoms may be extensions of corresponding severe symptoms, this study indirectly addressed that association. This study was not able to detect the association of HPV vaccine with extremely severe and rare events that do not have any relationship with the 24 reported symptoms. However, as we wrote in the methods sections, symptoms to be surveyed were chose in cooperation with the cooperation of the Aichi Branch of the All Japan Coordinating Association of HPV Vaccine Sufferers and reflect the symptoms they felt were associated with the HPV vaccine.

The third limitation was the use of self-reported symptoms without guidelines or diagnoses by a physician. Although we were unable to treat diseases based on a physician guidelines, the significance of this study derives from its design as an epidemiological study with internal comparison of vaccinated and unvaccinated participants. Outcomes were not diseases or syndromes but simple symptoms and their influence on daily life. Thus personal interpretation to questions is likely to be rather homogeneous despite the fact that the validity of the symptoms using self-reporting is unknown in this population. If we required a clinical diagnosis, there would have been more missing data, resulting in less precise and even more biased results. We decided not to collect the information on clinical diagnoses, since it was neither essential nor realistic for this type of study design.

The temporal relationship between vaccination and symptoms deserves mention as a potential limitation. We did not completely exclude symptoms that occurred before vaccination because only vaccinated participants had an immunization date and deleting those cases from the vaccinated groups would have violated the validity of comparison. Instead, we devised two time frames for both vaccinated and unvaccinated girls (i.e., the grand analysis in Table 3 and the subgroup analysis II in Table 6) and observed the changes in ORs. Symptoms prior to vaccination may provide “noise”, but the validity of comparison between vaccinated and unvaccinated was maintained. Every epidemiological study design has unique strengths and weaknesses. To more clearly determine the association of vaccination and symptoms, many studies of diverse design and objective are needed. Despite several limitations, we believe that the results of the study are a significant addition to our knowledge on the safety of HPV vaccination in Japan.

## 5 Conclusion

In this large-scale survey in Nagoya, Japan, we found that the HPV vaccines were not significantly associated with the occurrence of 24 reported symptoms, thus suggesting no causal association between the vaccines and reported symptoms or adverse events.
